# Exploring the association between brain-derived neurotrophic factor levels and longitudinal psychopathological and cognitive changes in Sardinian psychotic patients

**DOI:** 10.1192/j.eurpsy.2022.2333

**Published:** 2022-10-25

**Authors:** Ulker Isayeva, Mirko Manchia, Roberto Collu, Diego Primavera, Luca Deriu, Edoardo Caboni, Novella Iaselli, Davide Sundas, Massimo Tusconi, Federica Pinna, Pasquale Paribello, Maria Scherma, Claudia Pisanu, Anna Meloni, Clement C. Zai, Donatella Congiu, Alessio Squassina, Walter Fratta, Paola Fadda, Bernardo Carpiniello

**Affiliations:** 1 Unit of Psychiatry, Department of Medical Sciences and Public Health, University of Cagliari, Cagliari, Italy; 2 Division of Neuroscience and Clinical Pharmacology, Department of Biomedical Sciences, University of Cagliari, Cagliari, Italy; 3 Unit of Clinical Psychiatry, University Hospital Agency of Cagliari, Cagliari, Italy; 4 Department of Pharmacology, Dalhousie University, Halifax, Nova Scotia, Canada; 5 Tanenbaum Centre for Pharmacogenetics, Campbell Family Mental Health Research Institute, Centre for Addiction and Mental Health, Toronto, Ontario, Canada; 6 Department of Psychiatry, Institute of Medical Science, Laboratory Medicine and Pathobiology, University of Toronto, Toronto, Ontario, Canada; 7 Centre of Excellence “Neurobiology of Dependence”, University of Cagliari, Cagliari, Italy

**Keywords:** BDNF, biomarkers, complex disorders, longitudinal trajectories, schizophrenia

## Abstract

**Background and hypothesis:**

Schizophrenia spectrum disorders are among the most debilitating mental disorders and has complex pathophysiological underpinnings. There is growing evidence that brain-derived neurotrophic factor (BDNF) can play a role in its pathogenesis. The present study investigated the longitudinal variation of serum BDNF levels in a 24-month observational prospective cohort study of Sardinian psychotic patients and its relationship with psychopathological and cognitive changes. Furthermore, we examined whether genetic variation within the *BDNF* gene could moderate these relationships.

**Study design:**

Every 6 months, 105 patients were assessed for their BDNF serum levels, as well as for a series of psychopathological, cognitive, and social measures. We performed a targeted analysis of four tag single nucleotide polymorphisms within the *BDNF* gene that were selected and analyzed using polymerase chain reaction. Longitudinal data were analyzed using mixed-effects linear regression models.

**Study results:**

We observed a declining longitudinal trajectory of BDNF levels in psychotic patients in general, and in relation to the severity of depressive and negative symptoms. BDNF serum levels also declined in patients scoring lower in cognitive measures such as attention and speed of information processing and verbal fluency. The rs7934165 polymorphism moderated the significant association between verbal fluency and BDNF levels.

**Conclusions:**

These findings in patients from real-world settings suggest a plausible role of peripheral BDNF levels as a marker of illness burden in schizophrenia spectrum disorders.

## Introduction

Schizophrenia (SCZ) and schizoaffective disorders (SAD) are relatively heterogeneous psychiatric disorders characterized by an array of symptoms including delusions, hallucinations, psychomotor, social, and cognitive impairment [1]. SCZ affects approximately 1% of the population [1], whereas SAD has a lifetime prevalence of approximately 0.3% [2]. SCZ and SAD are among the most debilitating mental disorders. However, our comprehension of their pathophysiological underpinnings remains inadequate. Indeed, they have complex pathogenesis that involves the interaction of multiple biological, genetic, and environmental factors [3]. SCZ and SAD both demonstrate high heritability according to family, twin, and adoption studies [4, 5], indicating a major genetic contribution to the illness risk. In fact, in SCZ, genome-wide association studies (GWASs) have successfully identified genetic variants contributing to the risk of developing SCZ, with 287 distinct genomic loci associated with the disorder [6].

In this context, the brain-derived neurotrophic factor (BDNF) seemingly plays a relevant role [7]. BDNF is the most prevalent and extensively studied neurotrophin in the human central nervous system (CNS), and is able to cross the blood–brain barrier [8]. BDNF is a key regulator of a wide range of neurophysiological processes including neurogenesis, neuronal differentiation [9], synaptogenesis, and long-term potentiation [10]. Like other neurotrophins, BDNF is synthesized as a precursor form, prepro-BDNF, which is further cleaved to produce mature BDNF either intracellularly or extracellularly [11].

Even though a strong correlation between peripheral BDNF and BDNF levels in the CNS has been reported [12], studies measuring BDNF levels in the serum as a potential biomarker of SCZ have yielded controversial results. Several studies have found decreased peripheral levels of BDNF in SCZ patients including first-episode psychosis patients [13–15] and chronic patients that have been medicated for a substantial period of time [16, 17]. Nevertheless, some studies found no difference between serum BDNF levels of SCZ patients and those of healthy controls [18, 19] and some even found an increase [20]. Meta-analytical findings demonstrated that there was a substantial decrease in BDNF serum and plasma levels of SCZ patients in acute episodes, suggesting that decreased peripheral BDNF levels can be considered as a biomarker of disease activity [21].

The finding of a decline in BDNF levels of SCZ patients is consistently observed. However, little is known about the temporal trajectory, and the modulators, of this decline. Indeed, several factors, including genetic, treatment, and clinical moderators, might affect the peripheral levels of BDNF in SCZ patients. BDNF levels appear to be affected by the Val66Met (rs6265) polymorphism, a single nucleotide polymorphism (SNP) in the *BDNF* gene leading to valine (Val) for methionine (Met) substitution at codon 66 [22]. The Val66Met polymorphism has been associated with intracellular trafficking and activity-dependent secretion of mature BDNF as well as neurocognitive deficits [23–25]. While some studies have found an association between the functional Val66Met variant and peripheral BDNF levels [26, 27], others have not [22, 28]. Consistently, two meta-analyses looking at the association between *BDNF* Val66Met polymorphism and several neurocognitive phenotypes found no significant difference between carriers of Met allele and Val/Val homozygotes [29, 30].

Peripheral BDNF levels might also be affected by treatment factors, and psychopathological and cognitive changes. Cognitive impairments are widely observed in patients with SCZ, and together with symptom severity, they are central to the prediction of the clinical and functional outcomes in SCZ [31, 32]. Several studies have shown the strong correlation between reduced peripheral BDNF levels and impaired neurocognitive and psychopathology test scores [33, 34].

Overall, the analysis of peripheral BDNF levels and their relationship with clinical and treatment factors in SCZ spectrum disorders has provided inconsistent results. These discrepancies assume even higher significance in consideration of the possible clinical relevance of using BDNF as diagnostic/prognostic marker in psychiatric disorders. For instance, it is conceivable that patients with a more severe course of illness or with higher genetic predisposition for SCZ might have lower levels of BDNF compared with those with less severe presentation (or with less genetic loading). The present study sought to investigate the longitudinal variation of serum BDNF levels in a 24-month observational cohort study named Longitudinal Assessment of BDNF in Sardinian Psychotic patients (LABSP) [35]. Several aims were tested, primarily the assessment of the variation of BDNF serum levels over time and its relationship with psychopathological changes, cognitive function, and social functioning. We hypothesized that BDNF serum levels would decrease in association with longer and more severe clinical course as well as in association with other possible factors such as cognitive decline. Furthermore, we also examined if genetic variation (four tag SNPs) within the *BDNF* gene could moderate these relationships. Finally, as an aside, we performed discriminatory analysis of SCZ and SAD using Receiver Operating Characteristic (ROC) curve, expecting that BDNF serum levels could differentiate individuals affected by the two disorders.

## Methods and Materials

### Sample

An *a priori* power analysis was conducted using repeated measures and sample size (RMASS; Center for Health Statistics, Chicago, IL, USA) software and the results indicated that the sample size of 59 individuals was sufficient to achieve 90% statistical power to detect significant difference at *α* = 0.05. Our sample was comprised of 105 patients with psychosis treated at the community mental health center of the Unit of Psychiatry of the Department of Medical Science and Public Health, University of Cagliari and University of Cagliari Health Agency, Cagliari, Italy. A Structured Clinical Interview for DSM-IV-TR Axis I Disorders, Patient Edition (SCID-I/P) [36] was administered by trained mental health professionals to confirm the diagnosis of SCZ or SAD. To be considered eligible for participation in the LABSP study, patients had to be between 18 and 65 years old; diagnosed with SCZ or SAD according to DSM-IV-TR, and with absence of acute psychopathological manifestations for the past 6 months before recruitment. Patients were excluded from the study if they refused to provide consent; had acute psychopathological symptoms, major unstable medical illness, severe mental retardation, major neurological disorder or a previous head injury, current drug and alcohol dependence, or severe illness-related cognitive impairment which affected their ability to participate in the study. The study was approved by the University of Cagliari Health Agency Ethics Committee and the protocol followed the principles of the Declaration of Helsinki. Written informed consent was obtained from all the patients.

### Assessment procedures

Recruited patients were assessed and evaluated using various measures at five different waves. Clinical, cognitive and social performance measures, and blood samples of the patients were collected at the baseline (T0), and at four consecutive time points: 6 months (T1), 12 months (T2), 18 months (T3), and 24 months (T4). The details of the assessment and evaluation process including used measures and materials [35], as well as preliminary findings [37], have been previously published. General psychopathology, the severity of positive and negative symptoms, and clinical status of the patients were assessed using the original 30-item Positive and Negative Syndrome Scale (PANSS) [38] and Clinical Global Impression Scale for Schizophrenia (CGI-SCH) [39]. In addition, we applied the consensus five-factor model of PANSS (PANSS-FCTcr) [40] consisting of 20 items that are categorized into Positive, Negative, Disorganized/Concrete, Excited, and Depressed factors, because previous studies have shown that PANSS-FCTcr better characterizes the structure of PANSS data [40, 41]. The Brief Assessment of Cognition in Schizophrenia (BACS) scale [42] was used to evaluate changes in cognitive domains including verbal memory, working memory, reasoning, and processing speed. The evaluation of social functioning was carried out using Personal and Social Performance (PSP) scale [43] that has shown to be a valid and reliable measure for patients with SCZ [44, 45].

### Sample collection and measurement of BDNF

For the assessment of BDNF serum levels, the blood from each patient was drawn at the same time of the day (between 8:00 and 10:00 AM) at each visit. Collected blood samples were kept at room temperature for approximately 4 h to allow for clotting, after which they were centrifuged at approximately 1,000 × *g* for 15 min. All samples were immediately stored in small aliquots at −20°C until analyzed. Then, the serum BDNF levels were determined using a commercial human enzyme-linked immunoassay (ELISA) kit (Booster Immunoleader, CA, USA; Cat. N° EK0307) following the manufacturer’s instructions. This kit is used for the quantitative detection of human BDNF in cell culture supernatants, serum, and plasma with a high sensitivity of <2 pg/mL, the measuring interval of 31.2–2,000 pg/mL, and no detectable cross-reactivity with other relevant proteins. The absorbance was measured using a microplate reader (Thermo Scientific Multiskan FC MA, USA) set at 450 nm within 30 min after the final step of the kit procedure.

### Genetic analysis

We used the Tagger program implemented in the Haploview v4.2 to select SNPs in linkage disequilibrium (LD) (*r* ^2^ ≥ 0.8) and with a minor allele frequency threshold of 0.01. The genotyping of SNPs rs1519480, rs11030104, rs6265 (Val66Met), and rs7934165 was performed using TaqMan probes on demand (C_11592757_20, C_1751792_10, C_11592758_10, C_1197567_10, ThermoFisher Scientific) on a StepOne Plus instrument (ThermoFisher Scientific). The reaction mixture was prepared in a final volume of 10 μL consisting of 5 μL of MasterMix (2 times), 0.5 μL of Custom TaqMan® SNP Genotyping Assay (20 times) containing primers marked as VIC and FAM to discriminate between alleles, 1 μL of cDNA, and 3.5 μL of RNase-free water. Polymerase chain reaction was performed with the following conditions: 30 s 60°C, 10 min 90°C, and 40 cycles at 95°C for 15 s and 60°C for 1 min.

### Statistical analysis

We analyzed the longitudinal data using mixed-effects linear regression models (MLRM). MLRM was used particularly because it allows the observation of the effects and interaction of multiple independent variables on a dependent variable while considering repeated measures across participants [46]. To test our hypotheses, we regressed our predictor variables and fixed effects on our log-transformed serum BDNF data. In a preliminary step, PANSS, CGI-SCH, BACS, and PSP scale scores of each subject at each time point were separately regressed on BDNF data to analyze the relationship between them. We fitted regression models while adjusting for age and sex and checked for linearity and homoscedasticity by examining plots of residuals against fitted values. Lastly, *BDNF* gene polymorphisms were added to the MLRM as a covariate to examine the possible moderating effect. The longitudinal data were analyzed using the statistical programming language R [47]. All regression models were fitted using “lme4” package [48]. A significance level of *p* < 0.05 was considered after Holm–Bonferroni corrections for multiple comparisons. ROC analysis, with sensitivity, specificity, and predictive value analysis of the ability to discriminate between SCZ and SAD in relation to longitudinal BDNF levels was also applied.

## Results

### Sample characteristics

Table 1 summarizes the demographic and clinical characteristics of the patients that participated in this study. The sample consisted of 105 patients including 64 with a diagnosis of SCZ and 41 with SAD. The mean age of the sample at the baseline was 48.85 ± 10.45 years.

**Table 1.** Main demographic and clinical characteristics of LABSP sample.

Variable (continuous)	*N*	Mean	SD
BDNF serum levels, ng/ml	105	25.45	13.67
Age, years	105	48.85	10.45
Age of onset, years	105	21.77	9.30
Duration of illness, months	105	308.51	134.33
Age at first treatment, years	105	24.23	8.95
Duration of untreated illness, months	105	29.07	54.60
Antipsychotics, chlorpromazine equivalents, mg/day	103	378.92	272.03
Variable (categorical)	*N*	%	
Sex (male)	74	70.5	
Presence of family history of mental disorders	64	61.0	
Presence of family history of schizophrenia	31	29.5	
Presence of family history of bipolar disorder	8	7.6	
Presence of family history of major depressive disorder	19	18.1	
Presence of family history of anxiety disorders	10	9.5	
Diagnosis of schizophrenia (SCID-I)	64	61.0	
Diagnosis of schizoaffective disorder (SCID-I)	41	39.0	
Diagnosis of obsessive–compulsive disorder (SCID-I)	5	4.8	
Diagnosis of cluster A personality disorders (SCID-II)	2	1.9	
Diagnosis of cluster B personality disorders (SCID-II)	2	1.9	
Diagnosis of cluster C personality disorders (SCID-II)	2	1.9	
Diagnosis of personality disorder NOS (SCID-II)	1	1.0	

*Abbreviations:* BDNF, brain-derived neurotrophic factor; LABSP, Longitudinal Assessment of BDNF in Sardinian Psychotic patients; SCID-I, Structured Clinical Interview for DSM-IV Axis I Disorders; SCID-II, Structured Clinical Interview for DSM-IV Axis II Disorders; SD, standard deviation.

### Associations between psychopathological symptoms and serum BDNF levels

The MLRM analysis showed a statistically significant decline in BDNF levels over time (*Z* = −4.9, *p* = 9.02 × 10^−7^). As shown in Table 2, analysis of the relationship between scores of original three-subscale PANSS and longitudinal BDNF levels yielded no significant association. However, when we examined the association between serum BDNF levels and five-factor PANSS scores we found a significant relationship between longitudinal BDNF levels and negative factor (*Z* = −2.245, *p* = 0.025). MLRM found a significant relationship between CGI depressive symptoms and BDNF levels (*Z* = −2.796, *p* = 0.005). This association remained significant when we added age and sex as covariates (*Z* = −2.819, *p* = 0.009). The results also showed a significant association between CGI negative symptoms and serum BDNF levels (*Z* = −2.057, *p* = 0.039). In terms of serum BDNF levels and scores of positive symptoms subscale of CGI-SCH, the results did not show a significant relationship between them.

**Table 2.** Results of mixed effects linear regression models.

	Unadjusted models			Adjusted models^a^		
Independent variables	Estimated coefficient	*Z* value	*p* value	Estimated coefficient	*Z* value	*p* value
PANSS, total score	−0.001525	−1.198	0.231	−0.001686	−1.342	0.359
PANSS, positive symptoms	−0.0009322	−0.194	0.846	−0.001650	−0.342	0.912
PANSS, negative symptoms	−0.005553	−1.590	0.112	0.005246	−1.494	0.270
PANSS, general psychopathology	−0.002449	−1.083	0.279	−0.002637	−1.177	0.478
PANSS-FCTcr, positive factor	−0.0007404	−0.101	0.92	−0.001647	−0.224	0.895
PANSS-FCTcr, negative factor	−0.009772	−2.245	**0.0248**	−0.009337	−2.118	0.068
PANSS-FCTcr, disorganized/concrete factor	−0.008265	−1.089	0.276	0.008029	−1.016	0.406
PANSS-FCTcr, excited factor	−0.005327	−0.742	0.458	−0.005763	−0.794	0.772
PANSS-FCTcr, depressed factor	−0.005875	−0.646	0.518	−0.006804	−0.755	0.771
CGI-SCH, severity positive symptoms	0.008801	0.487	0.626	0.005490	0.294	0.89
CGI-SCH, severity negative symptoms	−0.048374	−2.057	**0.0397**	−0.049882	−2.072	0.077
CGI-SCH severity depressive symptoms	−0.058876	−2.796	**0.0052**	−0.059671	−2.819	**0.0096**
CGI-SCH severity cognitive symptoms	−0.034471	−1.819	0.0689	−0.0340535	−1.733	0.166
CGI-SCH, global severity	−0.037967	−1.498	0.127	−0.039493	−1.559	0.244
BACS, verbal memory	0.001736	0.154	0.878	−0.0003715	−0.032	1
BACS, digit sequencing task (number of correct responses)	0.004699	1.034	0.301	0.004188	0.906	0.730
BACS, digit sequencing task (longest sequence recalled correctly)	0.00972	0.739	0.46	0.008199	0.612	1
BACS, verbal fluency (controlled oral word association test)^b^	0.011660	3.228	**0.0013**	0.0113920	3.167	**0.003**
BACS, attention and speed of information processing (symbol coding)	0.004147	2.194	**0.0282**	0.004306	2.142	0.064
BACS, executive functions, Tower of London	0.004319	1.024	0.306	0.0037557	0.846	0.795
PSP, total score	0.002010	1.251	0.211	0.001436	1.206	0.456

*Note*: Significant *p* values in bold.

Abbreviations: BACS, Brief Assessment of Cognitive in Schizophrenia scale; CGI-SCH, Clinical Global Impression Scale for Schizophrenia; PANSS, Positive and Negative Syndrome Scale; PANSS-FCTcr, consensus five-factor model of PANSS; PSP, Personal and Social Performance scale.

a Adjusted for age and sex.

b BACS, verbal fluency (category instances) variable was not included in the table as there were no sufficient observations to support the model.

### Associations between cognition, social functioning, and serum BDNF levels

The analyses showed that the BDNF serum levels decreased in patients scoring lower on symbol coding (*Z* = 2.194, *p* = 0.028) and semantic fluency (*Z* = 3.228, *p* = 0.001) subscales of BACS. The association between semantic fluency and BDNF levels remained significant after correcting for age and sex (*Z* = 3.167, *p* = 0.003). An examination of the relationship between the measure of social functioning and longitudinal BDNF levels yielded no significant associations.

### Moderating effect of genetic variation

The moderating effect of the SNPs within *BDNF* gene was analyzed using genotypic and allelic effect models including additive, dominant, and recessive models (Supplementary Table 2). We found a significant moderating effect of rs7934165 on the relationship between BACS subscale for semantic fluency and serum BDNF levels when analyzed using the recessive model (*Z* = −2.359, *p* = 0.0367). This interaction effect remained significant after adjusting for age and sex (*Z* = −2.339, *p* = 0.0466).

### ROC curve

We performed ROC analysis (Supplementary Figure 1) to examine the ability of our model to discriminate between SCZ and SAD in relation to longitudinal BDNF levels. The area under the curve (AUC) was calculated to evaluate the overall accuracy of the diagnostic test in discriminating between SCZ and SAD patients. Optimal diagnostic cut-off value was 2.8303 with a sensitivity 65.2% and specificity of 50.4%. Algorithm of this model had an AUC of 57.1% (95% CI: 0.5183–0.624), which indicates poor diagnostic performance.

## Discussion

In this study, we sought to clarify whether longitudinal BDNF serum levels of psychotic patients were correlated with treatment-related, psychopathological, cognitive, and social changes. Previous analysis of LABSP data examined the impact of antipsychotics on BDNF serum levels and found a significant longitudinal increase in those treated with depot/long-acting injectables, but not oral antipsychotics [37]. Our current study had several main findings. First, we found an overall decline in the trajectory of serum BDNF levels over time (Supplementary Figure 3). The results revealed that this decline was more pronounced in patients with more severe depressive and negative symptoms. In addition, BDNF serum levels were more declined in patients with lower scores in two cognitive domains including speed of processing and verbal fluency. Finally, when we examined the possible moderating effect of genetic polymorphisms within *BDNF* gene on these statistically significant associations, we found that rs7934165 polymorphism had a significant moderating effect on the association between verbal fluency and BDNF serum levels.

As mentioned before, the reason for the temporal decline in BDNF serum levels is unknown, but it might be hastened by disease progression or associated with other factors such as drug treatments and severity of clinical symptoms. A meta-analytic study by Fernandes et al. [49] established that peripheral BDNF levels of SCZ patients were moderately decreased in comparison to healthy controls and the decline was associated with the temporal course of the disease. Results of a recent meta-analysis by Rodrigues-Amorim et al. [50] showed that BDNF levels of both drug-naïve and medicated SCZ patients were reduced throughout the disease course. Indeed, the reduced BDNF expression have also been associated with neuroinflammation [51], increased cortisol levels [52], while enriched environment has been shown to increase BDNF levels in psychiatric and neurodegenerative disorders [53]. Nevertheless, the interaction between these factors should be better understood.

We did not find an association between BDNF levels and psychopathological symptoms when measured by 30-item three-subscale PANSS. Consistent with our results, some studies also did not observe any significant association between PANSS scores and peripheral BDNF levels [15, 18, 54]. Considering that five-factor PANSS has shown to be better at representing the dimensional structure of PANSS data, we utilized this model for our analysis as well. Surprisingly, we found a significant relationship between negative factor of the five-factor scale and reduced serum BDNF levels. This is a rather interesting outcome, as we also found a significant association between the severity of depressive and negative symptoms and reduced BDNF serum levels when we regressed CGI-SCH subscales on BDNF data. To our best knowledge, this is the first longitudinal study assessing the relationship between BDNF levels of psychotic patients and the severity of psychopathological symptoms using CGI-SCH. In their recent clinical study, Fang et al. [55] observed a significant association between reduced plasma BDNF levels and depressive symptoms in SCZ patients. Similar results were observed in another study where a significant negative association between depressive symptoms and BDNF serum levels of chronic SCZ patients was found [56].

A possible explanation for the observed correlation between negative and depressive symptoms and peripheral BDNF levels with CGI-SCH and not PANSS might be because CGI-SCH could be a more reliable measure than the 30-item PANSS for monitoring the longitudinal course of psychopathology in SCZ and SAD. [57] The five-factor/20-item PANSS model have also demonstrated to be a better fitting model for the symptoms of SCZ [41, 58, 59]. which could explain the reason the association between negative factor and serum BDNF levels was detected by this model and not the entire 30-item PANSS scale.

Cognitive impairment is a core symptom of SCZ and a number of studies have found an association between neurocognitive deficits and peripheral BDNF levels [33, 60, 61]. We found a significant correlation between BDNF levels and two cognitive domains including symbol coding and semantic fluency as measured by BACS. A recent meta-analysis examining the relationship between neurocognitive deficits and BDNF levels revealed that higher peripheral BDNF levels were associated with better performance on reasoning and problem-solving tasks in people with SCZ [29]. There is growing evidence associating BDNF to cognitive dysfunctions in psychotic patients at different stages of the disease. According to these findings, it can be assumed that peripheral BDNF levels can be considered as a potential biomarker for neurocognitive deficits in psychosis.

This study did not show any significant association between serum BDNF levels and Val66Met polymorphism within the *BDNF* gene. Our findings are in agreement with the results of meta-analysis and GWAS analysis of the Sardinian sample conducted by Terracciano et al. [62], where no association was found between Val66Met polymorphism and serum BDNF levels.

We first observed a significant moderating effect of Val66Met on the relationship between BDNF levels and BACS subscale for symbol coding, as well as BDNF levels and CGI depressive symptoms. However, these associations did not survive correction for multiple comparisons. Nevertheless, we found that, when using recessive model, rs7934165 polymorphism within the *BDNF* gene moderated the significant association between serum BDNF levels and verbal fluency cognitive domain. SNPs in the *BDNF* gene have been previously linked to peripheral BDNF levels as well as psychopathological and cognitive aspects of SCZ [26, 27, 63, 64]. One possible interpretation of these findings is that the genetic variants are to some extent incorporating the effect of the predictors. This would be consistent with the putative influence exerted by genetic variation on BDNF levels.

Our results should be interpreted considering several limitations. A major limitation of this study is the lack of control group. In addition, the sample size for this study is moderate, especially regarding the analysis of genetic variations of the *BDNF* gene. However, this is compensated by the longitudinal design and the presence of five time points for assessment. Moreover, the sample was rather heterogeneous regarding the duration and stage of illness. Indeed, some patients were in the early years of their illness course while others had in some instances decades of clinical history. Hence, we tested the interaction effect of duration of untreated psychosis and duration of illness on BDNF levels but did not find any significant effect (Supplementary Table 3). Likewise, in a recent meta-analysis by Rodrigues-Amorim et al. [50], they regressed the duration of illness on serum BDNF levels and did not find a significant effect. Nonetheless, larger sample size and more homogenous sample will be required for future studies to overcome these limitations. The relatively small sample size did not make possible to perform subgroup analyses and led to the inclusion of a limited number of covariates in MLRM models to prevent saturation. Even though there is a substantial genetic overlap between SCZ and SAD [5], further research should be undertaken to explore the differences between these subgroups. In addition to the latter point, not all confounding variables could be added to the same model to avoid overfitting and saturation. Finally, even considering the longitudinal design, and the MLRM modeling applied, it is not possible to exclude that some of the time-varying variables were not entirely captured by our analysis.

Another limitation of our study is that only serum BDNF levels of the patients were collected, whereas plasma BDNF levels were not assessed. We are not sure about the extent to which serum BDNF reflects the processes in CNS. While some authors have proposed that plasma BDNF is a more reliable proxy of what happens in CNS [49], others consider serum BDNF to be a better correlate of cortical BDNF levels [65]. Finally, the inability of ELISA kits to distinguish between pro and mature BDNF is another limitation of our study. Unlike mature BDNF, pro-BDNF plays role in inducing apoptosis, reducing dendritic spines, and other processes that may contribute to long-time depression [66]. Being able to measure BDNF by differentiating between these two isoforms is essential as they may have opposing effects, and future studies should consider using newly developed specific mBDNF and pro-BDNF ELISA assays [67] when investigating the proposed associations.

## Conclusion

Even considering these limitations, our study identified a longitudinal trajectory of decline of BDNF levels associated with decline in some cognitive domains and higher severity of depressive and negative symptoms in patients affected by SCZ and SAD. These findings in a real-world patient sample suggest a plausible role of peripheral BDNF levels as a marker of illness burden in SCZ spectrum disorders.

## Data Availability

The data that support the findings of this study are available from the authors upon request in anonymized form.

## Supplementary material

For supplementary material accompanying this paper visit https://doi.org/10.1192/j.eurpsy.2022.2333. click here to view supplementary material
