# Supplementary material for: Exploring the association between brain-derived neurotrophic factor levels and longitudinal psychopathological and cognitive changes in Sardinian psychotic patients
Source: Eur Psychiatry. 2022 Oct 25;65(1):e71. doi: 10.1192/j.eurpsy.2022.2333 (PMC9677441; doi:10.1192/j.eurpsy.2022.2333)

**Supplementary Material.**

**Supplementary Table 1**. Main Demographic and Clinical Characteristics of LABSP Sample (full table)

| **Variable (continuous)** | **N** | **Mean** | | **SD** | |
| --- | --- | --- | --- | --- | --- |
| BDNF serum levels, ng/ml | 105 | 25.45 | | 13.67 | |
| Age, years | 105 | 48.85 | | 10.45 | |
| Education, years | 105 | 9.26 | | 3.23 | |
| Offspring, N | 105 | 0.34 | | 0.95 | |
| Age of onset, years | 105 | 21.77 | | 9.30 | |
| Duration of illness, months | 105 | 308.51 | | 134.33 | |
| Age at first treatment, years | 105 | 24.23 | | 8.95 | |
| Duration of untreated illness, months | 105 | 29.07 | | 54.60 | |
| Cigarettes smoked per day, N | 46 | 19.83 | | 9.80 | |
| Weight, Kg | 74 | 77.32 | | 18.76 | |
| Height, cm | 74 | 167.84 | | 8.83 | |
| BMI | 73 | 27.3 | | 6.8 | |
| Waist circumference, cm | 45 | 90.47 | | 18.37 | |
| Antipsychotics, chlorpromazine equivalents, mg/day | 103 | 378.92 | | 272.03 | |
| **Variable (categorical)** | N | | % | | |
| Sex (male) | 74 | | 70.5 | |  |
| Age class |  | |  | |  |
| 18-20 | 2 | | 1.9 | |  |
| 21-25 | 38 | | 36.2 | |  |
| 26-44 | 58 | | 55.2 | |  |
| 45-65 | 7 | | 6.7 | |  |
| Marital status |  | |  | |  |
| Single | 8 | | 7.6 | |  |
| Married/Cohabiting | 10 | | 9.5 | |  |
| Divorced | 2 | | 1.9 | |  |
| Widowed | 83 | | 79.0 | | |
| NA | 2 | | 1.9 | | |
| Presence of offspring | 19 | | 18.1 | | |
| Employment |  | |  | | |
| Employed | 7 | | 6.7 | | |
| Student | 1 | | 1.0 | | |
| Registered disabled civilian | 95 | | 90.5 | | |
| Unemployed | 2 | | 1.9 | | |
| Presence of smoking | 52 | | 49.5 | | |
| History of substance abuse | 28 | | 30.8 | | |
| Current use of substances | 5 | | 5.5 | | |
| Presence of family history of mental disorders | 64 | | 61.0 | | |
| Presence of family history of schizophrenia | 31 | | 29.5 | | |
| Presence of family history of bipolar disorder | 8 | | 7.6 | | |
| Presence of family history of major depressive disorder | 19 | | 18.1 | | |
| Presence of family history of anxiety disorders | 10 | | 9.5 | | |
| Clinical course |  | |  | | |
| Episodic with full remission | 2 | | 1.9 | | |
| Episodic with residual symptoms | 37 | | 35.2 | | |
| Chronic with or without periodical relapses | 65 | | 61.9 | | |
| NA | 1 | | 1 | | |
| Presence of hospital admissions | 93 | | 88.6 | | |
| Diagnosis of schizophrenia (SCID-I) | 64 | | 61.0 | | |
| Diagnosis of schizoaffective disorder (SCID-I) | 41 | | 39.0 | | |
| Diagnosis of obsessive-compulsive disorder (SCID-I) | 5 | | 4.8 | | |
| Diagnosis of cluster A personality disorders (SCID-II) | 2 | | 1.9 | | |
| Diagnosis of cluster B personality disorders (SCID-II) | 2 | | 1.9 | | |
| Diagnosis of cluster C personality disorders (SCID-II) | 2 | | 1.9 | | |
| Diagnosis of personality disorder NOS (SCID-II) | 1 | | 1.0 | | |
| Long-acting antipsychotic therapy | 24 | | 22.9 | | |

Abbreviations: LABSP, longitudinal assessment of BDNF in Sardinian psychotic patients; BDNF, brain-derived neurotrophic factor; SD, standard deviation; SCID-I, Structured Clinical Interview for DSM-IV Axis I Disorders (SCID-I); SCID-II, Structured Clinical Interview for DSM-IV Axis II Disorders (SCID-II).

**Supplementary Table 2.** Interaction Effects of *BDNF* Gene Polymorphisms on the Significant Relationships Between Clinical Variables and Longitudinal BDNF Serum Levels

| Unadjusted Models Adjusted Models* | | | | | | | |
| --- | --- | --- | --- | --- | --- | --- | --- |
| Genetic Model | Interaction Effect | Estimated Coefficient | Z value | P value | Estimated Coefficient | Z  value | P  value |
| Genotypic Effect | CGI-SCH neg * rs7934165AG  CGI-SCH neg * rs7934165GG  CGI-SCH neg * rs6265_Val66MetCT  CGI-SCH neg * rs6265_Val66MetTT  CGI-SCH neg * rs11030104AG  CGI-SCH neg * rs11030104GG  CGI-SCH neg* rs1519480CT  CGI-SCH neg * rs1519480TT  CGI-SCH dep * rs7934165AG  CGI-SCH dep * rs7934165GG  CGI-SCH dep * rs6265_Val66MetCT  CGI-SCH dep * rs6265_Val66MetTT  CGI-SCH dep * rs11030104AG  CGI-SCH dep * rs11030104GG  CGI-SCH dep * rs1519480CT  CGI-SCH dep * rs1519480TT  PANSS-FCTcr neg * rs7934165AG  PANSS-FCTcr neg * rs7934165GG  PANSS-FCTcr neg * Val66MetCT  PANSS-FCTcr neg * Val66MetTT  PANSS-FCTcr neg * rs11030104AG  PANSS-FCTcr neg * rs11030104GG  PANSS-FCTcr neg * rs1519480CT  PANSS-FCTcr neg * rs1519480TT  BACS vf * rs7934165AG  BACS vf * rs7934165GG  BACS vf * rs6265_Val66MetCT  BACS vf * rs6265_Val66MetTT  BACS vf * rs11030104AG  BACS vf * rs11030104GG  BACS vf * rs1519480CT  BACS vf * rs1519480TT  BACS sc * rs7934165AG  BACS sc * rs7934165GG | -0.019640  0.007011  -0.002029  -0.128493  -0.012728  -0.085754  -0.318269  -0.358878  0.010978  -0.064021  -0.087187  -0.195373  -0.078009  -0.133319  0.008520  -0.032705  -0.0165056  -0.0006924  -0.009100  -0.004740  -0.009723  -0.003059  -0.360668  -0.365281  -0.022116  -0.018360  -0.008075  0.006823  0.003428  -0.017147  -0.006866  0.005207  -0.006509  -0.000631 | -0.321  0.101  -0.039  -1.385  0.244  -0.997  -1.410  -1.596  0.200  -0.973  -1.812  -1.756  -1.615  -1.485  0.055  -0.213  -1.521  -0.052  -0.929  -0.199  -0.981  -0.162  -1.541  -1.561  -2.310  -1.564  -0.990  0.254  -0.414  -0.917  -0.141  0.107  -1.277  -0.092 | 1  1  1  0.831  1  1  0.553  0.553  1  1  0.350  0.350  0.451  0.451  1  1  0.641  1  1  1  1  1  0.672  0.672  0.083  0.1178  1  1  1  1  1  1  0.806  1 | -0.034035  0.003097  -0.005331  -0.120319  -0.015879  -0.090576  -0.3192419  -0.3644498  0.008873  -0.053916  -0.092358  -0.217964  -0.084587  -0.113167  0.0105572  -0.0344213  -0.018652  -0.003033  -0.010030  -0.009081  -0.010382  -0.008865  -0.3613380  -0.3652496  -0.020922  -0.018149  -0.008188  0.009621  -0.003259  -0.026490  -0.0073740  0.0048186  -5.387e-03  3.117e-05 | -0.555  0.045  -0.102  -1.267  -0.305  -1.057  -1.417  -1.624  0.163  -0.823  -1.933  -1.862  -1.762  -1.236  0.068  -0.224  -1.784  -0.236  -1.034  -0.369  -1.051  -0.471  -1.546  -1.563  -2.235  -1.588  -1.023  0.349  -0.401  -1.525  -0.151  0.099  -1.099  0.004 | 1  1  1  1  1  1  0.626  0.611  1  1  0.319  0.319  0.391  0.791  1  1  0.372  1  1  1  1  1  0.782  0.782  0.102  0.337  1  1  0.859  0.509  1  1  0.815  1 |
|  | BACS sc * rs6265_Val66MetCT  BACS sc * rs6265_Val66MetTT  BACS sc * rs11030104AG  BACS sc * rs11030104GG  BACS sc * rs1519480CT  BACS sc * rs1519480TT | 0.000334  -0.019668  -0.002861  0.005817  0.015080  0.016632 | 0.077  -2.126  -0.651  0.680  0.274  0.302 | 1  0.151  1  1  1  1 | -0.0009947  -0.0183423  -0.003708  0.003331  0.017705  0.017875 | -0.228  -1.656  -0.840  0.360  0.322  0.326 | 1  0.377  1  1  1  1 |
| Allelic Effect |  |  |  |  |  |  |  |
| Additive  Model | CGI-SCH neg * rs7934165  CGI-SCH neg * rs6265_Val66Met  CGI-SCH neg * rs11030104  CGI-SCH neg * rs1519480  CGI-SCH dep * rs7934165  CGI- SCH dep * rs6265_Val66Met | -0.005269  0.030608  0.030971  -0.060522  0.029138  0.085461 | -0.154  0.779  0.837  -1.297  0.886  2.129 | 1  0.870  0.656  0.389  0.7515  **0.06.** | -0.004097  0.032106  0.034981  -0.0634840  0.0260747  0.085373 | -0.119  0.815  0.935  -1.364  0.790  2.111 | 1  1  0.714  0.518  1  0.104 |
|  | CGI- SCH dep * rs11030104  CGI-SCH dep * rs1519480  PANSS-FCTcr neg * rs7934165  PANSS-FCTcr neg * Val66Met  PANSS-FCTcr neg * rs11030104  PANSS-FCTcr neg * rs1519480  BACS vf * rs7934165  BACS vf * rs6265_Val66Met  BACS vf * rs11030104  BACS vf * rs1519480  BACS sc * rs7934165  BACS sc * rs6265_Val66Met  BACS sc * rs11030104  BACS sc * rs1519480 | 0.07134  -0.035488  0.001821  0.006109  0.005641  -5.8 x 10^-3^  0.0101454  0.005084  0.005611  0.011332  0.001191  0.001671  -1.8 x 10^-5^  0.001748 | 1.965  -0.842  0.279  0.744  0.754  -0.586  1.737  0.682  0.808  1.377  0.357  0.448  -0.005  0.380 | 0.0988  0.800  1  0.859  0.688  1  0.247  1  0.983  0.505  1  1  1  1 | 0.070881  -0.0364105  0.002191  0.007012  0.006491  -0.0048057  0.0102454  0.005259  0.006195  0.0111390  0.001458  0.001763  0.0008846  0.0003673 | 1.932  -0.857  0.342  0.855  0.866  -0.494  1.769  0.713  0.900  1.360  0.432  0.455  0.249  0.079 | 0.137  1  1  0.949  0.773  1  0.308  1  1  0.696  1  1  1  1 |
| Dominant  Model | CGI-SCH neg * rs7934165  CGI-SCH neg * rs6265_Val66Met  CGI-SCH neg * rs11030104  CGI-SCH neg * rs1519480  CGI-SCH dep * rs7934165  CGI- SCH dep * rs6265_Val66Met  CGI- SCH dep * rs11030104  CGI-SCH dep * rs1519480  PANSS-FCTcr neg * rs7934165  PANSS-FCTcr neg * Val66Met  PANSS-FCTcr neg * rs11030104  PANSS-FCTcr neg * rs1519480  BACS vf * rs7934165  BACS vf * rs6265_Val66Met  BACS vf * rs11030104  BACS vf * rs1519480  BACS sc * rs7934165  BACS sc * rs6265_Val66Met  BACS sc * rs11030104  BACS sc * rs1519480 | -0.031115  0.130329  0.080757  -0.343123 0.05945  0.161195  0.100181  -0.017300  -0.011400  0.001071  -0.0008102  -0.363185  0.004656  -0.012281  0.014787  0.001760  -0.004534  0.019944  -0.007481  0.016066 | -0.562  1.436  0.969  -1.534  1.090  1.462  1.140  -0.114  -0.984  0.045  -0.044  -1.554  0.467  -0.457  0.814  0.036  -0.796  2.235  -0.908  0.293 | 1  0.302  0.665  0.344  0.552  0.288  0.509  1  0.778  1  1  0.458  1  1  1  1  0.809  0.0728  0.728  1 | -0.040121  0.116628  0.079297  -0.347643  0.046096  0.160996  0.084033  -0.016776  -0.011439  0.0016427  0.002834  -0.363740  0.006152  -0.014703  0.021629  0.001597  -0.003760  0.016811  -0.007580  0.017394 | -0.734  1.242  0.964  -1.555  0.840  1.360  0.946  -0.111  -1.003  0.067  0.153  -1.558  0.629  -0.540  1.309  0.033  -0.646  1.550  -0.866  0.317 | 1  0.64221  1  0.451  1  0.521  1  1  0.948  1  1  0.569  1  1  0.762  1  0.795  0.485  0.773  1 |
| Recessive  Model |  |  |  |  |  |  |  |
|  | CGI-SCH neg * rs7934165  CGI-SCH neg * rs6265_Val66Met  CGI-SCH neg * rs11030104  CGI-SCH neg * rs1519480  CGI-SCH dep * rs7934165  CGI- SCH dep * rs6265_Val66Met  CGI- SCH dep * rs11030104  CGI-SCH dep * rs1519480  PANSS-FCTcr neg * rs7934165  PANSS-FCTcr neg * Val66Met  PANSS-FCTcr neg * rs11030104  PANSS-FCTcr neg * rs1519480  BACS vf * rs7934165  BACS vf * rs6265_Val66Met  BACS vf * rs11030104  BACS vf * rs1519480  BACS sc * rs7934165  BACS sc * rs6265_Val66Met  BACS sc * rs11030104  BACS sc * rs1519480 | -0.009513  -0.019794  -0.026732  0.050799  -0.011526  0.09500  -0.086966  0.041953  -0.011308  -0.008323  -0.008675  0.005212  -0.021399  -0.007453  -0.004852  -0.012005  -0.005358  -0.000798  -0.001911  -0.001735 | -0.166  -0.402  -0.547  1.034  -0.222  -2.049  -1.907  0.901  -1.117  -0.884  -0.927  0.527  -2.359  -0.929  -0.601  -1.404  -1.083  -0.190  -0.457  -0.370 | 1  1  0.924  0.352  1  0.121  0.157  0.3674  0.792  1  0.761  0.678  **0.0367**  0.675  0.816  0.321  0.558  1  0.795  1 | -0.019245  -0.023853  -0.031063  0.0545943  -0.008423  -0.096152  -0.088972  0.044304  -0.013620-0.009687  -0.009798  0.0043962  -0.020607  -0.007586  -0.005151  -0.0121672  -0.004580  -0.001845  -0.003060  -0.0003604 | -0.343  -0.486  -0.639  1.119  -0.165  -2.070  -1.962  0.948  -1.414  -1.039  -1.059  0.454  -2.339  -0.964  -0.652  -1.449  -0.950  -0.435  -0.727  -0.077 | 1  1  1  0.526  1  0.154  0.149  0.687  0.480  0.896  0.869  1  **0.0466**  0.751  0.776  0.442  0.410  0.8935  0.467  1 |

Abbreviations: CGI-SCH, Clinical Global Impression Scale for Schizophrenia; BACS, Brief Assessment of Cognitive in Schizophrenia; PANSS-FCTcr, consensus five-factor model of Positive and Negative Syndrome Scale

Holm–Bonferroni corrections were used to adjust P values for multiple comparisons between models

*Adjusted for age and sex

**Supplementary Table 3.** Interaction Effects of Duration of Illness and Duration of Untreated Psychosis on Longitudinal BDNF Serum Levels

|  | Estimated Coefficient | Std. Error | Z value | P value |
| --- | --- | --- | --- | --- |
| Time | -1.055 x 10^-2^ | 9.744 x 10^-2^ | -0.108 | 0.914 |
| Illness duration | 4.612 x 10^-4^ | 3.280 x 10^-4^ | 1.406 | 0.160 |
| Time:Illness duration | -9.663 x 10^-6^ | 2.873 x 10^-4^ | -0.034 | 0.973 |
| Time | -1.414 x 10^-2^ | 4.404 x 10^-3^ | -3.210 | **0.0013** |
| Duration of untreated psychosis | 1.062 x 10^-3^ | 2.041 x 10^-3^ | 0.520 | 0.603 |
| Time: Duration of untreated psychosis | -8.072 x 10^-5^ | 1.653 x 10^-4^ | -0.428 | 0.669 |

**Supplementary Figures**

**Supplementary Figure 1** The receiver operating characteristic (ROC) curve for binary classification


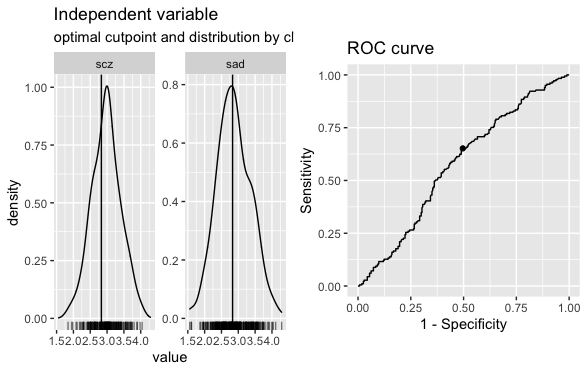


**Supplementary Figure 2** Raw BDNF levels at each wave of assessment with 95% confidence intervals (CI)


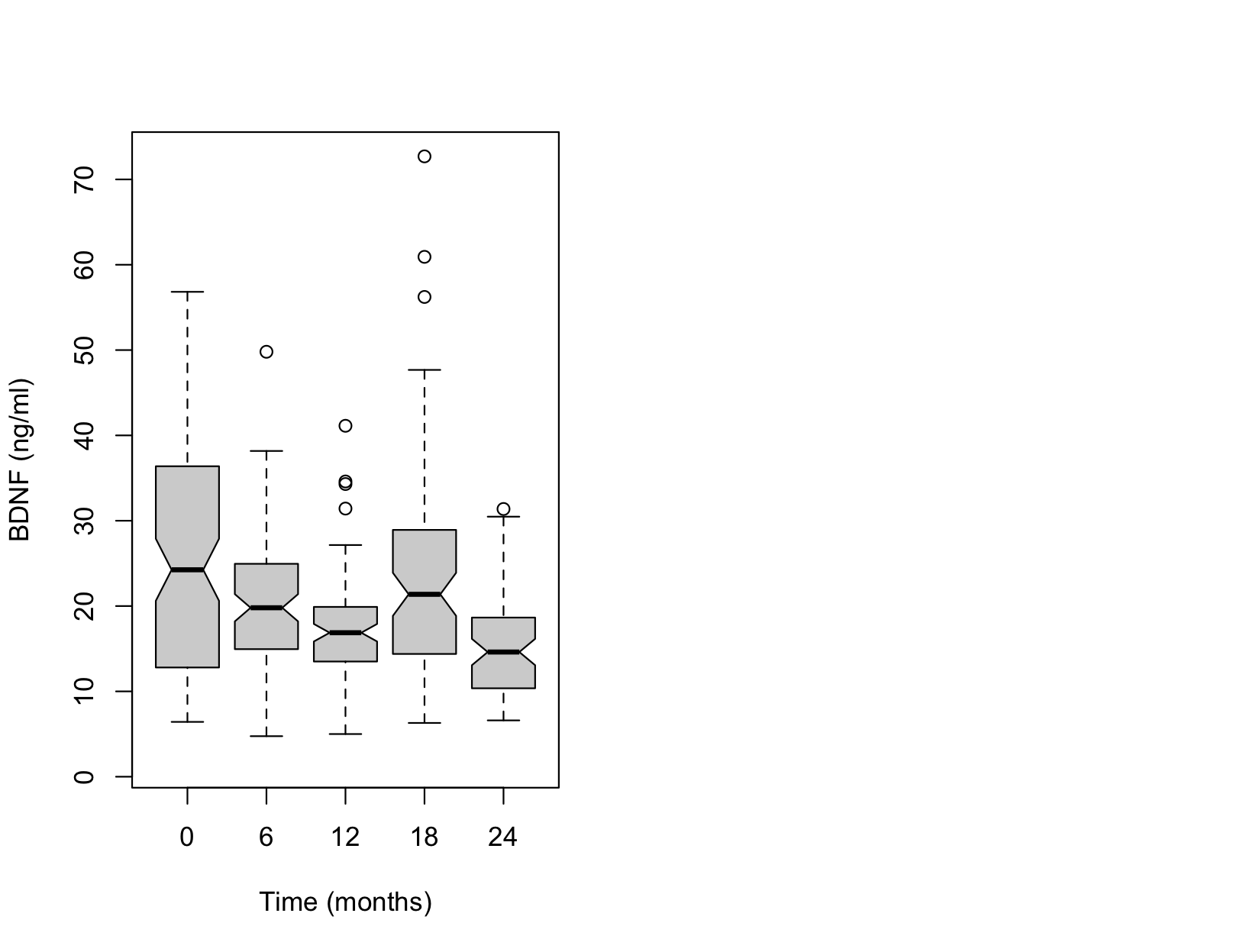


**Supplementary Figure 3** Log-transformed BDNF levels at each wave of assessment with 95% confidence intervals (CI)


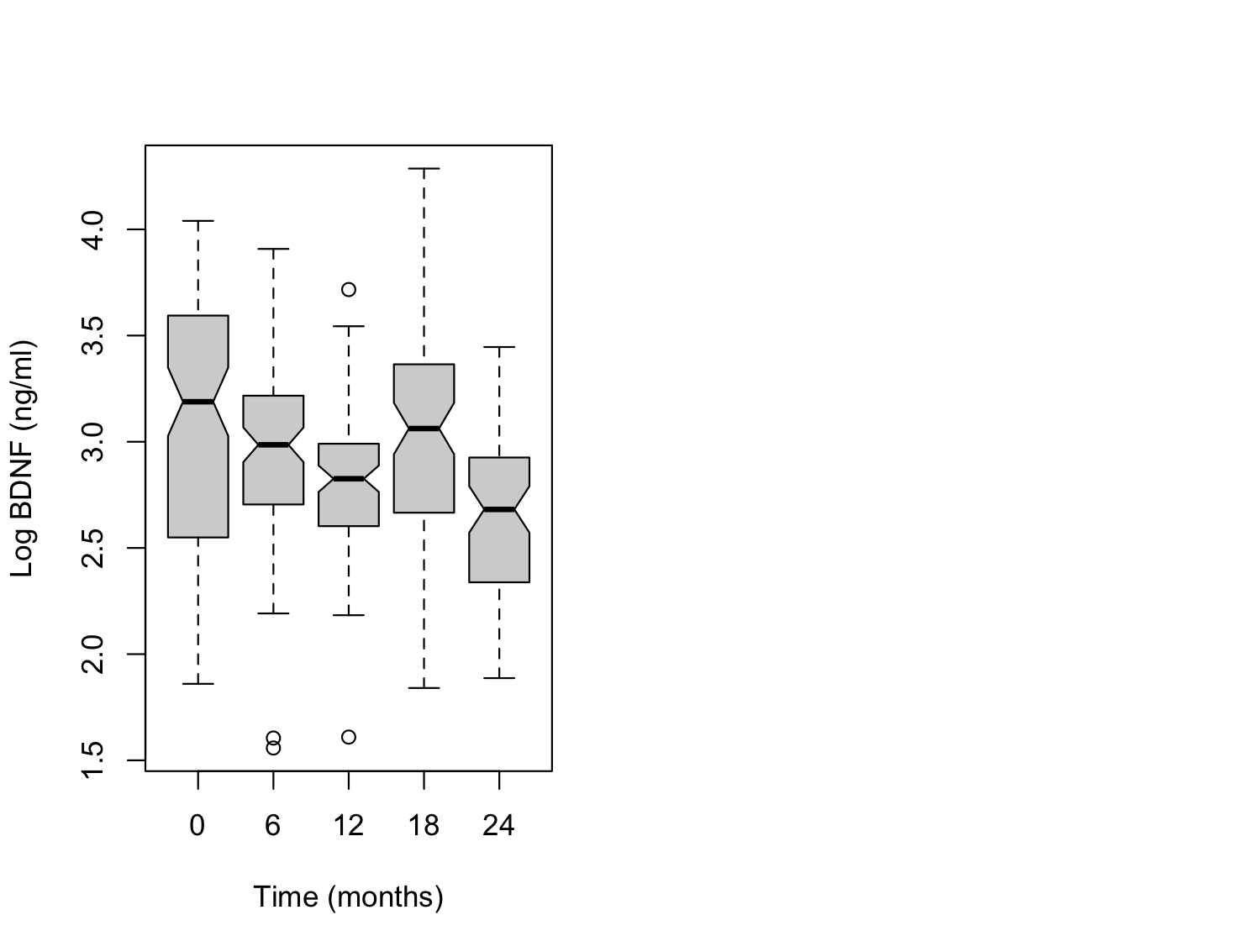

Supplement: Supplementary file 1 [file S0924933822023331sup001.docx]
